# Liposarcoma of the colon presenting as an endoluminal mass

**DOI:** 10.1186/1477-7819-7-78

**Published:** 2009-10-23

**Authors:** Marco D'Annibale, Maurizio Cosimelli, Renato Covello, Elisa Stasi

**Affiliations:** 1 Second Surgical Department, Regina Elena Cancer Institute, Via Elio Chianesi 53, Rome, Italy; 2 Department of Pathology, Histology and Cytodiagnosis, Regina Elena Cancer Institute, Via Elio Chianesi 53, Rome, Italy

## Abstract

**Background:**

Liposarcoma is one of the most common soft tissue sarcoma of adult life, usually occurring in the retroperitoneum and the extremities. Primary liposarcoma of the colon is very rare. The optimal treatment has not been established due to the small number of cases reported. We report a case of primary liposarcoma of the colon presenting as a massive intraluminal lesion.

**Case presentation:**

A 79-year-old woman presented with abdominal pain, progressive constipation and weight loss. A CT scan and a colonoscopy revealed an intraluminal mass in the transverse colon and multiple intraperitoneal lesions. The patient underwent surgical resection of the lesions. Pathologic examination was consistent with pleomorphic liposarcoma of the colon.

**Conclusion:**

Although no guidelines are available for the management of liposarcoma of the colon, surgical resection should be performed when feasible. Our patient's overall survival was satisfactory in spite of the multiple negative prognostic factors.

## Background

Liposarcoma is one of the most common soft tissue sarcomas of adult life. Histological classification includes five main subtypes: well-differentiated, myxoid, round cell, pleomorphic and dedifferentiated [1,2]. Liposarcoma usually occurs in deep soft tissues of extremities and retro peritoneum. A presentation primarily involving the gastrointestinal tract (GI) is unusual and liposarcoma of the colon is extremely rare. To our knowledge, only 8 cases have been reported to date [3-10]. We report a case of a primary locally advanced liposarcoma of the colon presenting as a massive intraluminal mass in the right colic angle.

## Case presentation

**Figure 1:**
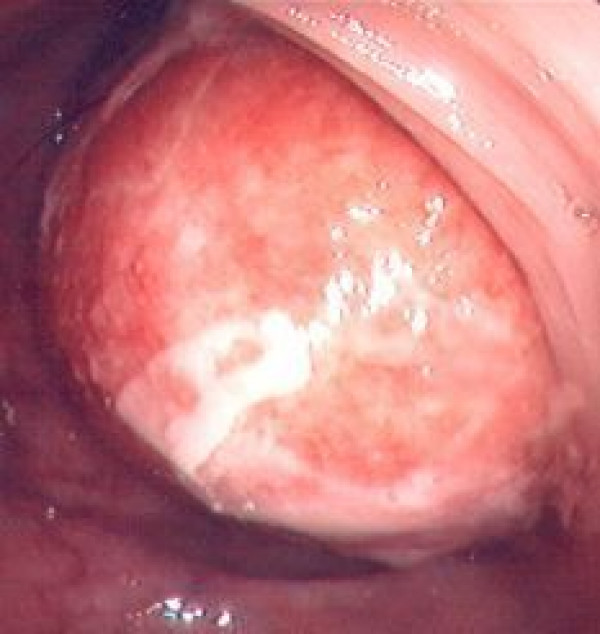
**Intraluminal mass visualized at colonoscopy**.

A 79-year-old woman, in good general conditions, presented with a 3-month history of episodic abdominal pain and progressive constipation associated with weight loss. Her past medical history and family history were unremarkable. Physical examination revealed a palpable, tender mass in the right upper quadrant of the abdomen. Laboratory tests on admission were within normal values, including tumour markers carcinoembryonic antigen (CEA), CA 125, CA 19.9 and CA 72.4. As a first approach, an abdominal ultrasonography (US) was performed, showing a hypoechoic ovoid mass in the right mesogastrium (max Ø 10 cm) and another hypoechoic round mass (max Ø 4.5 cm) in lumbo-aortic region. A computed tomography (CT) scan showed multiple intraperitoneal lesions: two solid, homogeneous masses located behind the proximal transverse colon, measuring respectively 9 and 6 cm, showing extensive vascularization following contrast injection; a solid homogeneous mass involving the distal transverse colon and left colic angle walls; multiple hypodense lesions with defined borders located in the hepatic hilum, Morrison's space, omentum and para-aortic region. A colonoscopy was performed, showing an intraluminal, yellowish lesion with clear margins in the transverse colon which didn't allow a further progression of the endoscope (Fig. 1). Biopsies showed unspecific inflammation: decreased density and dismorphism of glands, inflammatory infiltrate in corion, necrotic material and granulation tissue.

**Figure 2:**
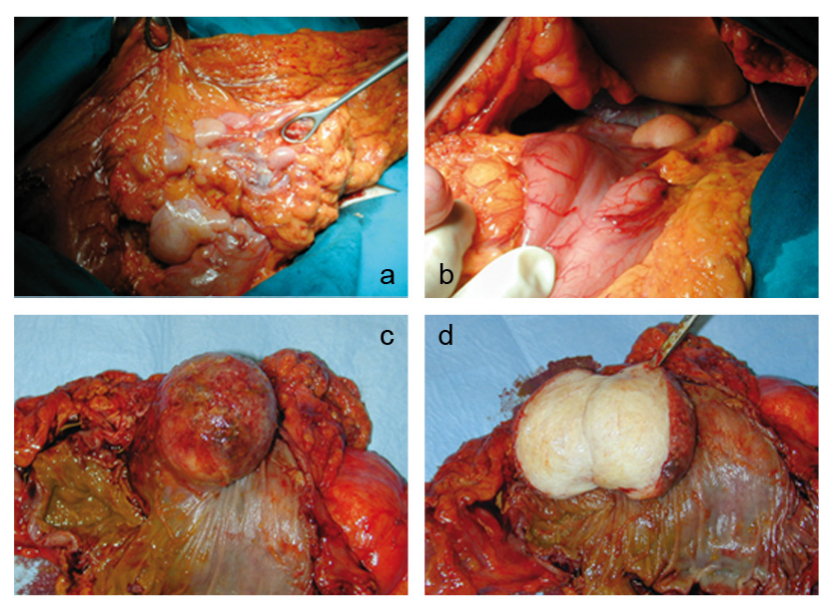
**Intraoperative findings of dissemination of the tumour at omentum (a) and stomach (b)**. Macroscopic view of the colon specimen showing an endoluminal mass, 50 × 52 × 47 mm in size, causing ulceration of the mucosal layer (c). Cut surface revealed a whitish, elastic-hard, encapsulated mass (d).

**Figure 3:**
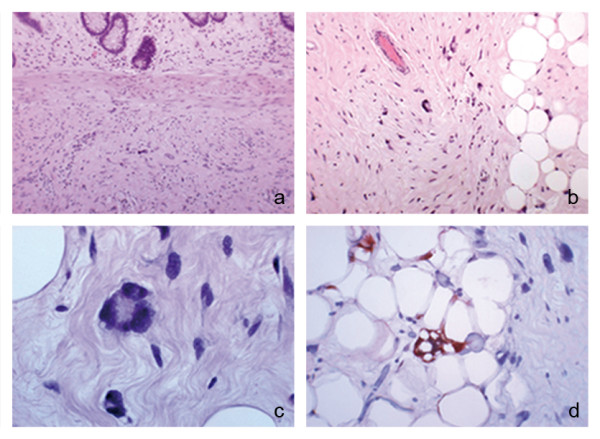
**Histologic examination of the primary tumour**. The tumour was mainly located in the submucosal layer. Cells with marked atypia in the centre of the field (hematoxylin-eosin, original magnification 10×); (b) Low power view of a sclerotic area with pleomorphic spindle shaped cells with marked atypia. In the centre of the field there is a multinucleated giant cell and some atypical, hypercromatic lipoblasts (hematoxylin-eosin, original magnification 20×); (c) High power view of a multinucleated giant lipoblast with floret-like features. This kind of cells is very similar to those found in the so called "malignant fibrous histiocytoma" (hematoxylin-eosin, original magnification 40×); (d) High power photomicrograph showing a lipoblast with a pleomorphic, enlarged, hypercromatic nucleus, and numerous cytoplasmic vacuoles containing fat, positive for S-100 (S-100 immunoistochemical stain, original magnification 40×).

Laparotomy revealed an intraluminal mass at the right colic angle and a dissemination of the tumour at stomach, omentum, mesentery and mesocolon (Fig. 2a-b). Frozen sections from transverse mesocolon and omentum were obtained during surgery, both showing adipose tissue fragments with marked cytologic alterations. There was no evidence of carcinomatous infiltration in the examined material. The patient underwent a right hemicolectomy, partial gastric resection, and excision of the omental and mesocolic masses. Removal of the mesenteric lesions was not attempted due to a massive dissemination of the disease at the origin of blood vessels. The patient recovered uneventfully and was discharged nine days after surgery. Gross examination of the colon specimen showed a whitish, elastic-hard, nodular, encapsulated mass 50 × 52 × 47 mm in size, mainly located in the submucosal layer, obliterating the colonic lumen and causing focal ulceration of the mucosal surface (Fig. 2c-d). Mucoid slimy areas were alternated with white and bright yellow areas. Focal areas of necrosis with a maximum size of 5 mm were also observed. The tumour was well circumscribed but not encapsulated; tumour borders were well defined with no evidence of infiltration of the mesocolon. Histological examination showed a highly undifferentiated tumour with pleomorphic spindle shaped cells with marked atypia mixed with multinucleated giant cells and atypical and multivacuolated lipoblasts, with bizarre, hypercromatic and scalloped nuclei (Fig. 3a-d). These findings were consistent with a diagnosis of pleomorphic liposarcoma of the colon; grade G3 according to FNCLL (French Fédération Nationale des Centres de Lutte Contre le Cancer) system [11]. The same findings were observed in all specimens. The mesenterial masses were respectively 32, 30, 25 and 25 mm smaller than the colonic mass. The masses were well circumscribed but not encapsulated, with well defined margins. The patient underwent a total dose of 54 Gy adjuvant radiation therapy 90 days after surgery, which resulted in a partial reduction of the residual lesions at the CT scan control performed six months afterwards. Follow-up protocol included clinical encounters and laboratory testing every three months, an upper abdomen ultrasound every six months, computed tomography every six months for the first year and annually thereafter, and annual endoscopic surveillance.

Twenty eight months after surgery, the patient developed progressive dysphagia with radiological evidence of tumour progression. Thirty months after surgery she died for an acute cardiac event. No autopsy was performed.

## Discussion

Liposarcomas of the large intestine usually present with nonspecific gastrointestinal symptoms such as bleeding, obstruction, diarrhea, abdominal pain and weight loss. It is thus difficult to distinguish them preoperatively from other colon cancers. Primary colonic liposarcoma tends to occur in adults, with a peak incidence between 5^th ^and 6^th ^decades, and an equal sex distribution [12]. WHO classification of liposarcomas recognizes five subtypes: well-differentiated, myxoid, round cell, pleomorphic and dedifferentiated. Pleomorphic liposarcoma is a high grade tumour containing a variable number of pleomorphic lipoblasts. It is aggressive, showing a high metastasis rate, with the lung representing the most common site of metastasis and a tumour associated mortality of 40% [13].

The GI tract does not represent a typical location for primary liposarcoma. However it can secondarily be involved as a site for metastases from liposarcomas located in the retroperitoneum and extremities [14,15]. Cases of primary liposarcoma have been reported in different parts of the GI tract, such as oesophagus, stomach, and small intestine [16-18]. Primary liposarcoma of the colon is a rare finding. The first case of primary colonic liposarcoma was reported by Wood and Morgenstern in 1989 [3]. Since then, seven other cases have been described [4-10] (Table 1). Patients were between 46 and 69 years of age, and 6 out of 8 were females. Clinical presentation included non-specific symptoms. Tumors were mostly located in the right colon. Histological subtypes included well differentiated, myxoid, pleomorphic and dedifferentiated types. A description of previously reported cases is shown in Table 1. According to the current criteria for histopathological diagnosis of liposarcoma [19], the 2 cases of atypical colonic lipomas reported by Snover in 1984 [20] can also be considered as well-differentiated liposarcomas of the colon. Recently a case of lipoleiomiosarcoma of the colon has been reported [21]. What is peculiar about our case is that the patient was 79, older than mean age of occurrence, and, with surgical evidence of residual disease, she had an overall survival of 30 months after diagnosis. Benign lipomas and low-grade liposarcomas have been demonstrated to dedifferentiate histologically into more aggressive subtypes [22,23]. It could be hypothesized that the patient had been affected by a low-grade lesion which progressively dedifferentiated to a pleomorphic liposarcoma.

**Table 1:** Review or reported cases of primary liposarcoma of the colon.

**Case report**	**Age/Sex**	**Presentation**	**Size/Location**	**Histological subtype**	**Extraluminal dissemination**	**Follow-up**
Wood and Morgenstern	62/F	Abdominal pain	7.5 × 8 × 12 cm, ileocecal valve	Myxoid	Yes	Died 2 yrs
Parks et al.	54/F	Abdominal discomfort, diarrhea, anemia, weight loss	6 × 4 × 5 cm, ascending colon	Pleomorphic	No	--
Magro et al	65/F	Abdominal pain, intussusception	5 cm, caecum	Well-differentiated	No	Alive 6 mo
Chen	52/F	Abdominal pain, hematochezia	7.5 × 5.5 × 5 cm, descending colon	Well-differentiated	Yes	Alive 2 yrs
Gutsu et al.	46/M	Abdominal pain	12 × 11 × 10 cm, ascending colon	Myxoid	No	Alive 1 yr
Shahidzadeh et al.	56/F	Hematochezia, anemia	3.5 × 3 × 2.8 cm, hepatic flexure	Well-differentiated	No	--
Chaudhary et al.	66/F	Abdominal pain, altered bowel habits, anemia, intussusception	4.5 × 3 × 2 cm, descending colon	Well-differentiated	No	Alive 10 mo
Jarboui et al.	69/M	Abdominal pain, constipation, weight loss	7 × 6 cm, splenic flexure	Dedifferentiated	Yes	Alive 10 mo
Current report	79/F	Abdominal pain, constipation, weight loss	5 × 5.2 × 4.7 cm, hepatic flexure	Pleomorphic	Yes	Died 2.5 yrs

The small number of cases reported has not allowed the determination of an appropriate diagnostic and therapeutic approach to this rare tumor. Complete surgical excision should be considered the gold standard. In our case, despite wide local excision was performed, the complete removal of the tumour was technically unfeasible due to the numerous extraluminal lipomatoid lesions. Our patient showed macroscopic evidence of residual disease and was thus candidate to further therapy. The role of chemotherapy in the management of liposarcoma has not been yet well established [24]. Conversely, radiotherapy has shown to affect survival rates [25]. Therefore, in consideration of the patient's age and clinical status, we decided to refer her to radiation therapy.

Prognosis for colonic liposarcoma is still difficult to predict. It may be affected by a variety of factors, including location, size, dissemination of the disease, as well as histological type [23,26]. No single morphological factor can predict the clinical outcome reliably; however, tumour depth size, more than 20 mitosis in 10 HPF (High Power Field), and areas of tumour necrosis are usually associated with a worse clinical prognosis [26,27]. In our case, despite evidence of multiple negative prognostic factors (dissemination of the disease in contiguous tissues, pleomorphic subtype and areas of necrosis), mitotic count was less than 10 mitosis in 10 HPF, which could justify the lower malignancy observed.

## Conclusion

Only a few cases of primary liposarcoma of the colon have been reported and optimal treatment strategies have not been established yet. However, surgery should be performed whenever feasible. The choice of therapeutic options should be based both on the natural history of the disease and on the patient's individual clinical status. The collection of a larger number of cases is needed for the definition of guidelines for the management of this rare tumour.

## Consent

Patient consent could not be obtained as the patient died. Written informed consent was obtained from the patient's son for publication of this case report and any accompanying images. A copy of the written consent is available for review by the Editor-in-Chief of this journal.

## Competing interests

The authors declare that they have no competing interests.

## Authors' contributions

MDA, the operating surgeon, conceived the design, collected data and participated in critical review. MC participated in data collection. RC assembled data and reviewed pathology. ES assembled data, reviewed literature and drafted the manuscript. All authors read and approved the final manuscript.

## References

[B1] Fletcher CD (2006). The evolving classification of soft tissue tumours: an update based on the new WHO classification. Histopathology.

[B2] Wu JM, Montgomery E (2008). Classification and pathology. Surg Clin North Am.

[B3] Wood DL, Morgenstern L (1989). Liposarcoma of the ileocecal valve: a case report. Mt Sinai J Med.

[B4] Parks RW, Mullan FJ, Kamel HMH, Walsh MY, McKelvey ST (1994). Liposarcoma of the colon. Ulster Med J.

[B5] Magro G, Gurrera A, Di Cataldo A, Licata A, Vasquez (2000). Well differentiated lipoma like liposarcoma of the caecum. Histopathology.

[B6] Chen KT (2004). Liposarcoma of the colon: a case report. Int J Surg Pathol.

[B7] Gutsu E, Ghidirim G, Gagauz I, Mishin I, Iakovleva I (2006). Liposarcoma of the colon: a case report and review of the literature. J Gastrointest Surg.

[B8] Chaudhary A, Arora R, Sharma A, Aggarwal S, Safaya R, Sharma S (2007). Primary colonic liposarcoma causing colo-colic Intusussception: a case report and review of literature. Gastrointest Cancer.

[B9] Shahidzadeh R, Ponce CR, Lee JR, Chamberlain SM (2007). Liposarcoma in a colonic polyp: case report and review of the literature. Dig Dis Sci.

[B10] Jarboui S, Moussi A, Jarraya H, Ben Mna K, Abdesselem MM, Kourda A, Ben Jilani S, Guettier C, Zaouche A (2009). Primary dedifferentiated liposarcoma of the colon: A case report. Gastroenterol Clin Biol.

[B11] Trojani M, Contesso G, Coindre JM, Rouesse J, Bui NB, de Mascarel A, Goussot JF, David M, Bonichon F, Lagarde C (1984). Soft tissue sarcomas of adults; study of pathological prognostic variables and definition of a histopathological grading system. Int J Cancer.

[B12] Weiss S, Goldblum J (2001). Enzinger and Weiss's soft tissue tumours.

[B13] Downes KA, Goldblum JR, Montgomery EA, Fisher C (2001). Pleomorphic liposarcoma: a clinicopathologic analysis of 19 cases. Mod Pathol.

[B14] Shibata Y, Sato K, Kodama M, Nanjyo H (2008). Metastatic liposarcoma in the jejunum causing intussusception: report of a case. Surg Today.

[B15] Monjazeb A, Stanton C, Levine EA (2004). Intussusception secondary to metastasis from a low-grade retroperitoneal liposarcoma. Am Surg.

[B16] Boggi U, Viacava P, Naccarato AG, Giulianotti PC, di Candio G, Battolla L, Mosca F (1997). Giant pedunculated liposarcomas of the esophagus: literature review and case report. Hepatogastroenterology.

[B17] Shokouh-Amiri MH, Hansen CP, Moesgaard F (1986). Liposarcoma of the stomach: a case report. Acta Chir Scand.

[B18] Mohandas D, Chandra RS, Srinivasan V, Bhaskar AG (1972). Liposarcoma of the ileum with secondaries in the liver. Am J Gastroenterol.

[B19] Dei Tos AP (2002). Atypical lipomatous tumour/well-differentiated liposarcoma. World Health Organization classification of tumours: pathology and genetics of tumours of soft tissue and bone.

[B20] Snover DC (1984). Atypical lipomas of the colon. Report of two cases with pseudomalignant features. Dis Colon Rectum.

[B21] Nahal A, Meterissian S (2009). Lipoleiomyosarcoma of the rectosigmoid colon. Am J Clin Oncol.

[B22] Weiss SW, Rao VK (1992). Well-differentiated liposarcoma (atypical lipoma) of deep soft tissue of the extremities, retroperitoneum and miscellaneous sites. A follow-up study of 92 cases with analysis of the incidence of "dedifferentiation". Am J Surg Pathol.

[B23] Reitan JB, Kaalhus O, Brennhovd IO, Sager EM, Stenwig AE, Talle K (1995). Prognostic factors in liposarcoma. Cancer.

[B24] Jones RL, Fisher C, Al-Muderis O, Judson IR (2005). Differential sensitivity of liposarcoma subtypes to chemotherapy. Eur J Cancer.

[B25] Strander H, Turesson I, Cavallin-Ståhl E (2003). A systematic overview of radiation therapy effects in soft tissue sarcomas. Acta Oncol.

[B26] McCormick D, Mentzel T, Beham A, Fletcher CD (1994). Dedifferentiated liposarcoma. Clinicopathologic analysis of 32 cases suggesting a better prognosis subgroup among pleomorphic sarcomas. Am J Surg Pathol.

[B27] Hornick JL, Bosenberg MW, Mentzel T, McMenamin ME, Oliveira AM, Fletcher CD (2004). Pleomorphic liposarcoma: clinicopathologic analysis of 57 cases. Am J Surg Pathol.

